# Up-regulation of lysophosphatidylcholine acyltransferase 1 (LPCAT1) is linked to poor prognosis in breast cancer

**DOI:** 10.18632/aging.102287

**Published:** 2019-09-18

**Authors:** Patrick Lebok, Aurelia von Hassel, Jan Meiners, Claudia Hube-Magg, Ronald Simon, Doris Höflmayer, Andrea Hinsch, David Dum, Christoph Fraune, Cosima Göbel, Katharina Möller, Guido Sauter, Frank Jacobsen, Franziska Büscheck, Kristina Prien, Till Krech, Rainer Horst Krech, Albert von der Assen, Linn Wölber, Isabell Witzel, Barbara Schmalfeldt, Stefan Geist, Peter Paluchoswski, Christian Wilke, Uwe Heilenkötter, Luigi Terracciano, Volkmar Müller, Waldemar Wilczak, Eike Christian Burandt

**Affiliations:** 1 Department of Pathology, University Medical Center Hamburg-Eppendorf, Hamburg D-20246, Germany; 2 General, Visceral and Thoracic Surgery Department and Clinic, University Medical Center Hamburg-Eppendorf, Hamburg D-20246, Germany; 3 Department of Pathology, Clinical Center Osnabrück, Osnabrück D-49076, Germany; 4 Breast cancer center, Niels-Stensen Clinic, Franziskus-Hospital Harderberg, Georgsmarienhütte D-49124, Germany; 5 Department of Gynecology and Clinic, University Medical Center Hamburg-Eppendorf, Hamburg D-20246, Germany; 6 Department of Gynecology, Regio Clinic Pinneberg, Pinneberg D-25421, Germany; 7 Department of Gynecology, Regio Clinic Elmshorn, Elmshorn D-25337, Germany; 8 Department of Gynecology, Regio Clinic and Senior Citizen Center Itzehoe, Itzehoe D-25524, Germany; 9 Cantonal Hospital Basel, University of Basel, Basel CH-4031, Switzerland

**Keywords:** breast cancer, LPCAT1, TMA, prognosis, immunohistochemistry

## Abstract

Dysregulation of lipid metabolism is common in cancer. Lysophosphatidylcholine acyltransferase 1 (LPCAT1) has been implicated with various cancer types. Here we analyzed by immunohistochemistry its expression in 2,197 breast cancers. LPCAT1 staining was found in 97.8% of 1,774 interpretable tumors, including 48.1% with weak, 28.7% with moderate, and 14.4% with strong expression. The frequency of LPCAT1 positivity depended on the histological tumor type. Moderate or strong LPCAT1 positivity was more common in cancers of no special type (NST) (46.2%) than in lobular carcinomas (25.9%; p<0.0001). Strong LPCAT1 was associated with BRE grade, tumor cell proliferation and overall survival in all cancers and in the subgroup of NST cancers (p<0.0001, each). In the subset of NST cancers the prognostic effect of LPCAT1 expression was independent of pT, and BRE grade (p<0.0001 each). A comparison with molecular features showed that LPCAT1 was strongly associated with estrogen receptor negativity (p<0.0001), progesterone receptor negativity (p<0,0001), amplification of HER2 (p<0.0001) and MYC (p=0.0066), as well as deletions of PTEN (p<0.0001) and CDKNA2 (p=0.0151). It is concluded that LPCAT1 overexpression is linked to adverse tumor features and poor prognosis in breast cancer. These data also highlight the important role of lipid metabolism in breast cancer biology.

## INTRODUCTION

Breast cancer is the most common cancer in females worldwide and is also the leading cause of cancer-related deaths in the female population [1]. Surgical removal of the cancer is the standard therapy. Whether adjuvant systemic treatment is performed or not depends on the individual risk situation. The histological grade, tumor size and presence of lymph node metastasis are basic parameters to assess the prognosis of individual patients. Additional molecular analyses are increasingly employed but still not sufficient to reliably determine tumor aggressiveness [2, 3]. The analysis of further molecular properties could eventually improve the reliability of prediction of tumor aggressiveness.

Previous studies have described activation of lipid biosynthesis and lipid remodeling to occur commonly in cancer cells [4]. The phospholipid biosynthesis/remodeling enzyme lysophosphatidylcholine acyltransferase 1 (LPCAT1) is a key enzyme in the lipid-remodeling pathway known as Lands cycle [5]. LPCAT1 has a physiological role in the lung where it generates the dipalmitoyl phosphatidylcholine component of pulmonary surfactant [6, 7], in non-inflammatory platelet-activation factor remodeling pathway [8] and in retinal photoreceptor homeostasis [9]. Overexpression of LPCAT1 was recently described in colorectal cancer [10], prostate cancer [11], lung cancer [12] and clear cell renal cell carcinomas [13]. LPCAT1 overexpression led to a significant growth advantage in cultured colorectal cancer cells [10].

A recent study on a cohort of 80 patients has suggested that up-regulation of LPCAT1 in breast cancer may contribute to tumor progression and predict early tumor recurrence [14]. To broaden our knowledge on LPCAT1 as a biomarker in breast cancer we tested 2,197 breast cancer samples for LPCAT1 expression and analyzed associations with histologically and molecularly defined cancer subgroups as well as follow-up information. Our data identify and validate high LPCAT1 expression as a strong prognostic biomarker for early tumor recurrence in breast cancer.

## RESULTS

### Technical issues

A total of 1,774 (80.7%) of 2,197 tumor samples were interpretable in our TMA analysis. Non-informative cases (473 spots; 19.3%) were due to missing tissue samples or the absence of unequivocal cancer tissue in the TMA spot.

### LPCAT1 expression in normal breast tissue and breast cancer

**Figure 1:**
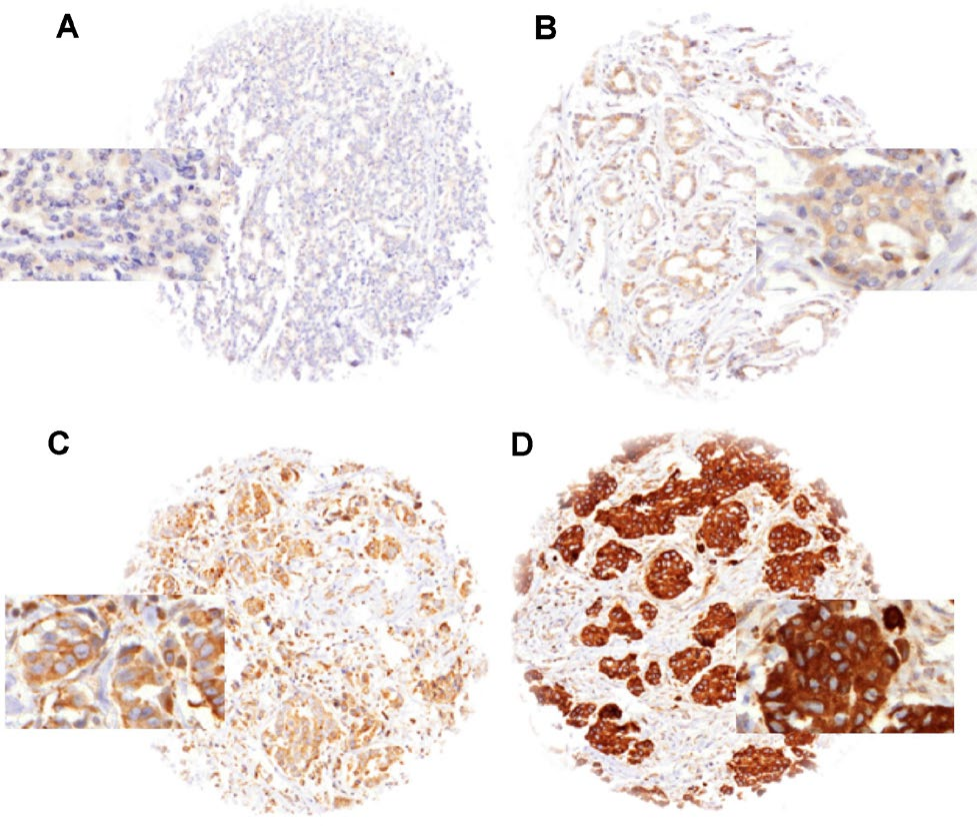
LPCAT1 staining in breast cancer with (**A**) negative, (**B**) weak, (**C**) moderate and (**D**) strong staining.

Normal breast tissues showed moderate to strong LPCAT1 expression in luminal cells under the selected experimental conditions. In cancer, detectable LPCAT1 immunostaining was seen in 1,619 of our 1,774 (91.3%) tumors and was considered weak in 48.1%, moderate in 28.7% and strong in 14.4% of tumors. A fraction of 8.7% showed no detectable LPCAT1 staining and was classified as negative. Representative images of LPCAT1 immunostainings are shown in Figure 1. LPCAT1 expression varied between histological breast cancer subtypes (Table 1). Strong LPCAT staining was for example more often seen in papillary (18.5%) and NST (16.2%) than in lobular carcinomas (5.7%; p<0.0001 for lobular vs. NST cancers). Strong LPCAT staining was also seen in some rare breast cancer subtypes including carcinoma with apocrine differentiation (5 of 12 strongly positive), carcinoma with medullary features (11 of 60) and glycogen-rich clear cell type (3 of 13).

**Table 1:** LPCAT1 immunostaining and breast cancer phenotype.

	**LPCAT1 IHC result (%)**					**P**
	**N**	**Negative**	**Weak**	**Moderate**	**Strong**	
All cancers	1774	8.7	48.1	28.7	14.4	
**Histology**						<0.0001
No special type (NST)	1277	7.8	46.0	30.0	16.2	
Lobular carcinoma	228	11.4	62.7	20.2	5.7	
Medullary carcinoma	60	6.7	35.0	40.0	18.3	
Cribriform carcinoma	53	22.6	50.9	18.9	7.5	
Tubular carcinoma	38	18.4	57.9	23.7	0.0	
Papillary carcinoma	27	11.1	37.0	33.3	18.5	
Mucinous carcinoma	50	2.0	54.0	34.0	10.0	
Other and rare types*	41	4.9	39.0	29.3	26.8	
**Tumor stage**						0.0176
pT1	638	9.2	52.2	27.9	10.7	
pT2	856	7.9	46.6	28.6	16.8	
pT3	101	10.9	47.5	22.8	18.8	
pT4	202	8.4	42.6	34.2	14.9	
**Nodal status**						0.2865
pN0	768	9.0	50.0	28.0	13.0	
pN1	656	8.2	47.7	29.3	14.8	
pN2	102	9.8	37.3	33.3	19.6	
**BRE grade**						<0.0001
1	415	14.5	59.8	22.2	3.6	
2	668	7.8	54.2	27.4	10.6	
3	570	4.7	35.4	34.4	25.4	
**ER status**						<0.0001
Negative	406	5.9	32.0	33.3	28.8	
Positive	1289	9.7	52.8	27.2	10.3	
**PR status**						<0.0001
Negative	1056	8.7	45.9	27.7	17.7	
Positive	567	9.2	52.2	29.5	9.2	
**HER2 status**						<0.0001
Normal	1153	9.1	51.2	28.4	11.3	
Amplified	245	5.7	31.4	29.0	33.9	

*including carcinoma with apocrine differentiation and metaplastic carcinoma of no special type.

### Association with tumor phenotype and molecular features

High levels of LPCAT1 immunostaining were significantly linked to unfavorable tumor features including high pT stage, high BRE grade, estrogen and progesterone receptor negativity, and HER2 amplification (p<0.0001 each). This was also seen for the subgroup of NST carcinomas (Table 1). The comparison of LPCAT1 expression with previously described other genomic alterations such as c-MYC- amplification [15] as well as deletions of PTEN [16–18] and CDKNA2 [19, 20] revealed significant associations with LPCAT1 up-regulation (p<0.02; Table 2). LPCAT1 expression was unrelated to MDM2 amplifications (p=0.67).

**Table 2:** LPCAT1 staining and genomic alterations.

		**LPCAT1 IHC (%)**				**P**
		**Negative**	**Weak**	**Moderate**	**Strong**	
**PTEN**	Normal (n=906)	9.1	49.6	28.8	12.6	
	Deleted (n=220)	5.9	35.5	29.1	29.5	≤0.0001
**c-MYC**	Normal (n=992)	9.9	48.4	28.7	13.0	
	Gain (n=258)	6.6	42.2	31.0	20.2	
	Amplification (n=64)	3.1	42.2	31.3	23.4	0.0066
**CDKN2A**	Normal (n=832)	8.8	47.1	29.1	15.0	
	Deleted (n=151)	7.3	37.1	30.5	25.2	0.0151
**MDM2**	Normal (n=1469)	8.5	47.9	28.9	14.8	
	Amplified (n=94)	9.6	43.6	27.7	19.1	0.6727

### Association with tumor cell proliferation

Data on tumor cell proliferation as evaluated by KI67 immunohistochemistry were available from a previous study [19]. These were correlated with the present LPCAT1 staining. The mean Ki67LI increased from 23.5 ±1.2 for LPCAT1 negative cancers to 37.3 ±1.0 for cancers with strong LPCAT1 expression (p<0.0001; Table 3). This association was also seen in all tumor subsets with identical pT stage, nodal, ER, PR and HER2 status, BRE grade (only in grade 2 and 3).

**Table 3:** LPCAT1 expression and Ki67-labeling index.

	**LPCAT1**	**N**	**Ki67LI**					**LPCAT1**	**N**	**Ki67LI**		
**All cancers** p<0.0001	Negative	135	23.5	±	1.2		**BRE grade 2** p<0.0001	Negative	46	22.7	±	1.1
	Weak	717	24.7	±	0.5			Weak	304	23.1	±	0.7
	Moderate	432	30.0	±	0.7			Moderate	157	24.6	±	0.8
	Strong	219	37.3	±	1.0			Strong	61	30.6	±	0.8
**pT1** p<0.0001	Negative	50	20.7	±	1.8		**BRE grade 3** p<0.0001	Negative	25	35.2	±	2.9
	Weak	258	21.1	±	0.8			Weak	170	34.8	±	1.1
	Moderate	148	25.9	±	1.1			Moderate	158	40.2	±	1.2
	Strong	56	34.9	±	1.7			Strong	124	42.6	±	1.3
**pT2** p<0.0001	Negative	57	23.8	±	2.0		**ER neg.** p<0.0001	Negative	22	34.0	±	3.4
	Weak	340	27.1	±	0.8			Weak	116	32.1	±	1.5
	Moderate	200	32.8	±	1.1		Moderate	115	38.8	±	1.5
	Strong	120	38.4	±	1.4		Strong	103	43.3	±	1.6
**pT3** p=0.0253	Negative	10	23.1	±	5.2		**ER pos.** p<0.0001	Negative	110	21.7	±	1.2
	Weak	41	27.1	±	2.5			Weak	568	23.6	±	0.5
	Moderate	19	33.7	±	3.7			Moderate	303	26.7	±	0.7
	Strong	18	39.3	±	3.8			Strong	110	31.7	±	1.2
**pT4** p=0.0035	Negative	17	30.2	±	3.2		**PR neg.** p<0.0001	Negative	82	24.2	±	1.6
	Weak	76	25.2	±	1.5			Weak	431	25.2	±	0.7
	Moderate	60	29.7	±	1.7			Moderate	252	32.2	±	0.9
	Strong	25	36.4	±	2.7			Strong	168	39.9	±	1.2
**pN0** p<0.0001	Negative	56	20.6	±	1.9		**PR pos.** p=0.0435	Negative	48	22.4	±	1.9
	Weak	311	23.9	±	0.8			Weak	243	24.4	±	0.8
	Moderate	183	29.5	±	1.1			Moderate	152	27.5	±	1.0
	Strong	88	37.6	±	1.5			Strong	41	27.9	±	2.0
**pN+** p<0.0001	Negative	58	24.6	±	1.9		**HER2 norm.** p<0.0001	Negative	89	24.2	±	1.5
	Weak	297	25.8	±	0.8			Weak	497	24.6	±	0.6
	Moderate	186	30.9	±	1.1			Moderate	277	29.5	±	0.9
	Strong	97	37.0	±	1.5			Strong	110	36.8	±	1.4
**BRE Grade 1** p=0.1291	Negative	50	18.5	±	1.5		**HER2 amp.** p=0.0072	Negative	14	30.4	±	3.6
	Weak	201	17.8	±	0.7			Weak	63	33.1	±	1.7
	Moderate	78	20.7	±	1.2			Moderate	61	36.2	±	1.7
	Strong	12	22.2	±	3.0			Strong	73	40.1	±	1.6

### Prognostic significance of LPCAT1 expression

**Figure 2:**
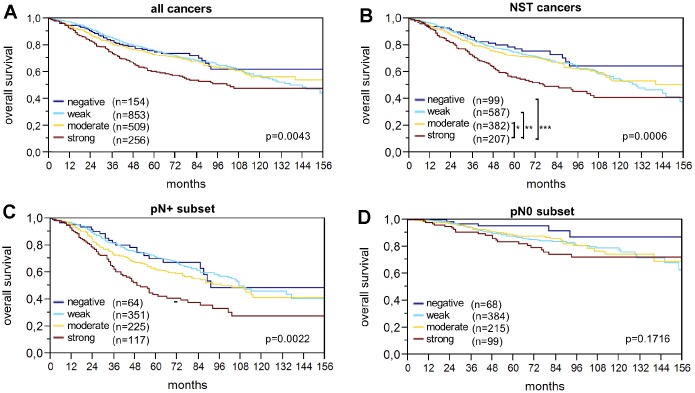
LPCAT1 staining and overall survival in (**A**) all cancers, (**B**) the no special type (NST) cancer subset, (**C**) nodal positive, and (**D**) nodal negative cancers.

Raw survival data were available for 1,774 cancers with interpretable IHC results. Strong LPCAT1 expression was closely associated with shortened overall survival (p=0.0043; Figure 2A). The association between strong LPCAT1 expression and poor prognosis was even more pronounced in the subgroup of NST cancers (p=0.0006; Figure 2B) and in the nodal positive subset (p=0.0022; Figure 2C) but was not seen in nodal negative cancers (p=0.1716; Figure 2D). Multivariate analysis for NST cancers including pT stage, nodal status, BRE grade and hormone receptors did not identify LPCAT1 expression as an independent prognosticator of survival (Table 4).

**Table 4:** Cox proportional hazards for survival of established prognostic parameter in breast cancers of no special type.

**Variable**	**Subset**	**N**	**HR (95% CI)**	**P**
**ER**	Negative vs. positive	1998	1.5 (1.2-2.0)	≤0.0001
**PR**	Negative vs. positive	1907	1.2 (1.0-1.6)	0.1158
**HER2 IHC**	3 vs. 0	1984	1.2 (0.8-1.5)	0.4753
**BRE grade**	3 vs. 1	2008	2.1 (1.5-2-9)	≤0.0001
**pT stage**	4 vs. 1	2161	2.3 (1.7-3.3)	≤0.0001
**pN stage**	2 vs. 0	1820	5.6 (3.9-8.0)	≤0.0001
**LPCAT1 expression**	Strong vs. neg./weak/mod.	1772	1.2 (0.8-1.4)	0.7550

“N” gives the total number of all cases with survival data in each category. Hazard ratios (HR), confidence intervals (CI) and P-values correspond to the indicated subsets.

ER (estrogen receptor); PR (progesterone receptor); HR (hazard ratio); CI (confidence interval).

## DISCUSSION

Our immunohistochemical analysis showed positive LPCAT1 staining in 91.2% of tumors, including 14.4% with strong staining and 76.8% with weak to moderate staining. Since the staining intensity of LPCAT1 in normal breast glands was usually moderate, these data suggest that LPCAT1 is overexpressed in about 15% of breast cancers. Our data are consistent with a previous study by Abdelzaher and Mostafa comparing LPCAT1 expression in 80 breast cancers of NST and 30 non-neoplastic epithelial breast tissues [14]. In this study, LPCAT1 immunostaining was found to be higher in tumor tissue than in non-neoplastic epithelial breast tissue.

Significant differences between the individual breast cancer subtypes fit well with the recognized biological differences between tumor entities. However, the most striking result of our study is the strong association of LPCAT1 expression with an unfavorable histological phenotype and clinical outcome. These data are also confirmed by the results of Abdelzaher and Mostafa [14], which describe links between LPCAT1 immunostaining and high-grade, advanced TNM stage, T stage and lymph node stage in their series of 80 breast cancer patients. It is noteworthy that the 12% of cancers classified as “strong LPCAT1 expressers” behaved significantly worse than the other cancers, especially when the clinical outcome (overall survival) was taken into account. A striking correlation between strong LPCAT1 expression and unfavorable clinical outcome was also found in more homogeneous cancer subtypes, such as 1,277 cancers of no special type and 758 nodal-positive cancers, suggesting possible clinical applicability of LPCAT1 measurement for prognostic evaluation. That LPCAT1 overexpression is also observed in aggressive forms of a broad variety of other cancer types [21–23] suggests a general role of this protein during tumor progression. Based on its molecular function as a key enzyme of lipid synthesis in the Land’s cycle it is believed that LPCAT1 up-regulation reflects a consequence of the increased demand for lipid-depending cellular structures such as membranes and fatty acids in rapidly proliferating tumor cells (reviewed in [24]). This is also supported by work demonstrating that inhibition of enzymes of the Land’s cycle limits the growth of cancer cells and reduces tumorigenesis in various tumor cell models [25].

The molecular database attached to our TMA enabled us to study the relationship of LPCAT1 expression with molecular features that had earlier been analyzed on the same TMA. According to our study, expression levels of LPCAT1 were strongly associated with amplifications of HER2 and MYC, negative ER and PR status and PTEN deletions, which are all linked to adverse tumor features and poor patient outcome. Virtually all of these molecules are implicated in the regulation of processes depending on sufficient supply of lipids and fatty acids, such as cell growth and proliferation, and some of them are known to directly contribute to rate limiting steps of lipid metabolism. For example, there is emerging evidence that PTEN controls lipid biosynthesis via its downstream target Maf1 [26]. MYC has been shown to cooperate with sterol regulatory element-binding protein 1 (SREBP1), a transcription factor involved in regulating lipid homeostasis that was shown to promote epithelial-mesenchymal transition in colon cancers [27] and ER regulates expression of at least 20 genes involved in fatty acid metabolism in breast cancer [28].

Activation of lipid metabolism in tumor cell proliferation is widely accepted. This fits well with the strong correlation of Ki67 expression level with high LPCAT1 expression seen in this study. That this relationship retained high statistical significance in various analyzed cancer subgroups defined by an identical status of morphologic or molecular parameters argues for a particular strong role of LPCAT1 for tumor growth. Apart from the need of producing lipids for dividing cells, LPCAT1 expression could also impact tumor cell proliferation by the production of metabolic intermediates for synthesis of cellular signaling molecules [29]. Moreover, Lipid metabolism has been associated with cellular proliferation and energy storage (reviewed in [30], similar to the prominent Warburg effect [29]. It has been suggested that increased lipid metabolism is either needed to support the growth of rapidly dividing cells, or to maintain elevated glycolysis (the Warburg effect). Various tumors undergo exacerbated endogenous lipid metabolism irrespective of the levels of extracellular lipids [31].

It is a limitation of our study that no data on therapy are available. The rate of LPCAT1 positivity may be higher in case of heterogeneity since we had only one 0.6 mm spot per cancer analyzed. Many data were taken from previous analyses of the same TMA, some of which have been performed more than 10 years ago. This study is an excellent example for how the TMA technology facilitates the development of molecular databases that can be used for every new study.

In summary, our data identify LPCAT1 expression as a prognostic biomarker with potential clinical utility in breast cancer. It appears well possible, that LPCAT1 measurement, either alone or in combination, may be utilized for better clinical decision-making in the future. The findings also highlight the potentially important role of lipid metabolism in breast cancer as a parameter for tumor aggressiveness.

## MATERIALS AND METHODS

### Patients

A preexisting tissue microarray (TMA) with 2,197 human breast cancer samples from paraffin-embedded tissue specimens fixed in 4% neutral buffered formalin was used [19]. The samples were consecutively collected between 1984 and 2000. The median patient’s age was 63 years (range 25–101). Raw survival data were available from 1,982 patients. The mean follow-up time was 63 months (range 1–176). The TMA was produced as describe earlier in detail [32]. In short, one 0.6 mm core was taken from a representative cancer tissue block from each patient. The samples were distributed across 6 TMA blocks. A control area containing 20 samples of normal breast tissue was present on each TMA block. Four μm sections of the TMA blocks were transferred to an adhesive coated slide system (Instrumedics Inc., Hackensack, New Jersey) for IHC analysis. The TMA was annotated with data from previous FISH studies for HER2, MYC, CCND1, p53, PTEN, CDKN2A and MDM2 as well as IHC studies on estrogen receptor (ER), progesterone receptor (PR) expression and Ki67-labeling index (Ki67LI) [19, 20, 33, 34].

### Immunohistochemistry

Freshly cut TMA sections were immunostained in a single experiment. Slides were deparaffinized and exposed to heat-induced antigen retrieval (5 min, 121°C in Tris-EDTA-citrate buffer at pH 7.8). Primary antibody specific for LPCAT1 (rabbit, Protein Tech; at 1/1350 dilution was applied at 37°C for 60 minutes. Bound antibody was then visualized using the EnVision Kit (Dako, Glostrup, Denmark) according to the manufacturer´s directions. LPCAT1 staining was found on the membrane and in the cytoplasm of positive cells. Evaluation of the immunohistochemical staining was performed as previously described [35]. In brief, tumors with complete absence of staining were scored as “negative”. Cancers with a staining intensity of 1+ in up to 70 %, or 2+ in ≤ 30 % of the tumor cells were scored as “weak”. A “moderate” score was given to cancers with a staining intensity of 1+ > 70 %, 2+ in up to 70 %, or 3+ in ≤30 % of tumor cells. The score was considered “strong” if staining intensity was 2+ in >70 % of tumor cells or 3+ in > 30 % of tumor cells.

### Statistics

Contingency tables and chi-square test were calculated to find associations between LPCAT1 expression and clinico-pathological variables. Anova and F-test was applied for associations between LPCAT1 expression and Ki67LI. Kaplan-Meier curves and the log-rank tests were applied to test for differences between stratified survival functions. Cox proportional hazards regression analysis was performed to test for independence and significance between pathological and molecular variables. JMP 12.0 software (SAS Institute Inc., NC, USA) was used.

### Ethics approval

The Ethics Committee of the Ärztekammer Hamburg approved the study protocol (WF-049/09). According to local laws (HmbKHG §12a), patient informed consent was not required. Patient records/information were anonymized and de-identified prior to analysis. All procedures have been performed in compliance with the principles outlined in the Helsinki Declaration.
